# Automatic Tooth Segmentation of Dental Mesh Based on Harmonic Fields

**DOI:** 10.1155/2015/187173

**Published:** 2015-08-27

**Authors:** Sheng-hui Liao, Shi-jian Liu, Bei-ji Zou, Xi Ding, Ye Liang, Jun-hui Huang

**Affiliations:** ^1^School of Information Science and Engineering, Central South University, Changsha 410083, China; ^2^Department of Stomatology, First Affiliated Hospital of Wenzhou Medical University, Wenzhou 325000, China; ^3^Department of Stomatology, Xiangya Hospital of Central South University, Changsha 410008, China; ^4^Xiangya Stomatological Hospital of Central South University, Changsha 410008, China

## Abstract

An important preprocess in computer-aided orthodontics is to segment teeth from the dental models accurately, which should involve manual interactions as few as possible. But fully automatic partition of all teeth is not a trivial task, since teeth occur in different shapes and their arrangements vary substantially from one individual to another. The difficulty is exacerbated when severe teeth malocclusion and crowding problems occur, which is a common occurrence in clinical cases. Most published methods in this area either are inaccurate or require lots of manual interactions. Motivated by the state-of-the-art general mesh segmentation methods that adopted the theory of harmonic field to detect partition boundaries, this paper proposes a novel, dental-targeted segmentation framework for dental meshes. With a specially designed weighting scheme and a strategy of a priori knowledge to guide the assignment of harmonic constraints, this method can identify teeth partition boundaries effectively. Extensive experiments and quantitative analysis demonstrate that the proposed method is able to partition high-quality teeth automatically with robustness and efficiency.

## 1. Introduction

In recent years, much effort has been spent in developing computerized systems for clinical and research applications in dentistry. Most computerized algorithms for orthodontic diagnostic and treatment require 3D dental mesh models, which are often needed to extract, move, and rearrange teeth for simulation of the treatment outcome. Thus, teeth segmentation is an important step in many automated and semiautomated, computer-based dental software packages.

However, tooth segmentation on dental meshes remains a difficult task [1]. Dental meshes from patients often have teeth crowding problems when adjacent teeth are misaligned, thus making the interstices between them irregular and difficult to distinguish. Various tooth shapes make outlining tooth contours difficult. Artifacts resulting from scanning or model-making errors on commonly obtained clinical meshes make teeth segmentation more challenging.

General mesh segmentation approaches are not directly suited for segmenting dental meshes because they lack adjustments to handle complex tooth shapes and teeth arrangements. Other segmentation approaches proposed to handle dental meshes have shortcomings, such as being either labor-intensive or not sufficiently accurate [2]. Although several commercial products in this field are available, such as “3Shape,” their user interactions are intensive and significantly influence the accuracy of results.

Many tooth segmentation methods prefer to use surface curvature when identifying potential tooth boundaries, as they follow the most widely cited mesh segmentation criterion, the minima rule, which states that human perception usually divides a surface into parts along the concave discontinuity of the tangent plane [3, 4]. However, surface curvature estimation is typically error-prone for real-world noisy models; for example, curvature distribution on clinical dental models is generally complicated and irregular (see Section 2 for further description).

This paper aims to develop an automatic and robust method for segmentation of digital dental models. Motivated by state-of-the-art general interactive mesh segmentation methods [5–8], we developed a convenient and efficient dental segmentation framework, which identifies tooth boundaries by a dental-targeted, harmonic field. This approach has three major benefits.

1. The dental-targeted harmonic field is robust to various tooth shapes, complex malocclusion, and crowding problems and is insensitive to noise with minimum parameter considerations.
2. The dental-targeted harmonic field can guarantee closed tooth boundary extraction from dental mesh, unlike curvature-based methods needing complex connectivity and morphologic operations in most cases.
3. The whole dental segmentation procedure is automatic, which requires no user interaction to generate teeth with accurate and high-quality cutting boundaries.

## 2. Related Work

This paper focuses on dental mesh segmentation. We introduce general mesh segmentation methods before dental specific methods. The theory of harmonic field will be given at the end of the section.

### 2.1. General Segmentation Method

Numerous mesh segmentation approaches have been proposed in computer graphics. Some of these algorithms are automated, like clustering [9], random walk [10], shape diameter function [11], fitting primitives [12], and fast marching watershed [13]. Most of these methods aim to partition different regions based on similarity measures [14], but defining a semantic subarea of tooth with various shapes remains a challenging task and none of these methods is directly suitable for our specific application.

Recently, sketch-based interactive mesh segmentation methods have become very popular [5–8]. Most of these methods employ harmonic field theory and similar user interfaces, but they are different in computational weighting scheme and constraint styles. Interested readers should refer to literature such as surveys of comparisons between these sketch-based methods [15]. These methods have good performance in general mesh segmentation but require time-consuming and error-prone user interaction when dealing with special and complicated tooth shapes and arrangements. Furthermore, they cannot produce high-quality cutting boundaries in cases where no obvious concave regions are near the location of interaction.

While our method also employs the harmonic field for segmentation, it fully exploits the dental characteristics (see Section 3.3), which makes the proposed method smart, accurate, and more robust in tooth partition.

### 2.2. Dental-Targeted Segmentation Method

A large group of dental segmentation approaches prefer to use surface curvature to identify tooth boundaries. The typical routine can be summarized as follows.

(1) Curvature estimation: principal curvatures, including minimum principal curvature [16–19] and mean principal curvature [1], are used to measure the surface property quantitatively. (2) Teeth-part rough locating: though not always necessary, this is a plane-estimating technique, based on PCA that produces a cutting plane to separate gingiva and teeth part, introduced in previous works [1, 20]. We also involve this step in our framework, but the method we used is more convenient and effective (see Section 3.2). (3) Thresholding: a curvature threshold value is inevitably needed to separate potential tooth boundary regions from the rest when using curvature fields for boundary identification. That value can be obtained either interactively, such as when using an intuitive slider [16], or by taking a result of an experiential equation or even plugging in a constant preset number [19, 20]. In fact, a global threshold value used in this mandatory step is one of the major drawbacks; it can significantly influence this entire solution, because the thresholding generally cannot appropriately distinguish target objects from the rest of the region, leading to either over- or undersegmentation. (4) Potential boundary region refining: because the curvature field introduces lots of useless features in tooth crown regions and is sensitive to noise in identifying tooth boundary methods [1, 17, 19], using a morphologic operation will further refine the region obtained by thresholding. For some complicated dental models, however (e.g., in cases of adjacent teeth crowding when the interstices in between are irregular and difficult to distinguish), the potential tooth boundary region may still be incomplete even after morphologic operation. (5) Boundary locating and refining: as proposed in previous works [1, 16, 17, 19], a skeleton operation is used to extract boundaries from potential regions. However, teeth with such boundaries are unacceptable for precise clinical treatment planning. Refinements should be carried out to make sure the boundary of each tooth is both smooth and precise [1]. More seriously, the extracted boundaries could be incomplete (e.g., opened) so special refinement should be taken to close the opened boundaries before attempting smooth and precise operations. All such procedures make the entire framework tedious and complicated.

Kondo et al. introduced a fully automatic algorithm to segment tooth from dental models using two range images [20]. However, they used a rectangular inspection spoke to cut the model, which will introduce inaccurate cuts for severe malocclusions. Kronfeld et al. proposed a snake-based approach that starts with an initial contour on the gingiva and evolves through a feature attraction field [21]. The cusps of each tooth are then selected to start a local tooth contour and evolve until each tooth bottom is reached. The approach is automatic but may not produce good results when the model has boundary noises that interrupt a feature field defined by the curvature information.

As another option, interactive dental partition methods [22, 23] allow users to select several boundary points interactively for segmentation. Geodesics are then generally used to connect two adjacent control points. The interactive partition procedures are intuitive and capable of segmenting complicated dental models, but the shortcomings are also quite obvious; for example, users would have to rotate or translate multiple times to carefully specify particular mark points to generate one accurate tooth boundary. Such tedious and time-consuming experiences certainly are undesirable and impracticable for clinical application.

Recently, Zou et al. proposed an interactive tooth partition method based on harmonic field [24]. The method has great flexibilities which benefit from their specially designed user interfaces. But the employed harmonic field is only “tooth-target”; in other words, only one tooth could be identified manually one time, and the whole dental mesh has to be processed multiple times to finish the whole segmentation. In contrast, our novel segmentation framework is able to segment all teeth only once under a uniform harmonic field automatically, which greatly improves the partition efficiency.

### 2.3. Harmonic Field

In mathematics, a harmonic field on 3D mesh, *M* = (*V*, *E*, *F*), is a scalar field attached to each mesh vertex and satisfies ΔΦ = 0, where *V*, *E*, and *F* denote vertex, edge, and face set of *M*, respectively. The symbol, Δ, is the Laplacian operator, subject to particular Dirichlet boundary constraint conditions. For example, 0 and 1 are used as minimum and maximum constraints in most harmonic field computations. The standard definition of Laplacian operator on a piecewise linear mesh, *M*, is the operator:

(1) $$\begin{array}{c} \Delta \Phi_{i}=\underset{(i,j)\in E}{\sum }w_{ij}\left(\Phi_{i}-\Phi_{j}\right), \end{array}$$

where *w*_*ij*_ is a scalar weight assigned to the edge, *E*_*ij*_. This Poisson equation, ΔΦ = 0, can be solved by the least-squares sense, which leads to

(2) $$\begin{array}{c} A\Phi =b,\\ A=\left[\begin{array}{c} L\\ C \end{array}\right],\\ b=\left[\begin{array}{c} 0\\ b^{\prime } \end{array}\right], \end{array}$$

where *L* is the Laplacian matrix given by

(3) $$\begin{array}{c} L_{ij}=\left\{\begin{array}{cc} \underset{k\in N_{1}\left(i\right)}{\sum }w_{ik}, & \text{if }i=j,\\ -w_{ij}, & \text{if }\left(i,j\right)\in E,\\ 0, & \text{otherwise}, \end{array}\right. \end{array}$$

where *N*_1_(*i*) is the 1-ring neighbor set of vertex, *i*. *C* and *b*′ are matrix and vector, respectively, standing for the constraints in the harmonic field. Different weighting scheme and constraints will lead to a different kind of harmonic field. For example, let *α*_*ij*_ and *β*_*ij*_ denote angles opposite to *E*_*ij*_, respectively; the standard cotangent-weighting scheme will be given by

(4) $$\begin{array}{c} w_{ij}=\frac{\text{cot}\alpha_{ij}+\text{cot}\beta_{ij}}{2}, \end{array}$$

leading to a smooth, transiting harmonic field, suited to applications like mesh deformation [25] and direction field design [26]. However, the standard weighting scheme cannot identify the local shape variation; this drawback makes it no longer suitable for segmentation purposes.

Our method utilizes a novel, dental-targeted weighting scheme, which preserves the nice property of classic cotangent-weighting scheme, while having the ability to sense the concave feature for each tooth boundary. A special constraint assigning strategy is also proposed accordingly.

## 3. Materials and Methods

Since human teeth occur in different shapes and their arrangements vary substantially from one individual to another, careful selection of test datasets is necessary in tooth segmentation studies. We evaluate 60 sets of dental models including both low and up jaw of varying complexity and precision. For instance, teeth on some of the dental meshes have severe malocclusion and crowding problem, while others may be absent from the jaw. These datasets were accumulated in the years between 2010 and 2014, at Xiangya Hospital of Central South University and the First Affiliated Hospital of Wenzhou Medical University, from patients who need medical treatments such as orthodontics or dental implantation. These models are acquired by a 3D dental scanner or intraoral scanner with accuracy of 0.01 mm–0.1 mm. Each of the dental meshes is guaranteed to be manifold and nondegenerate as preprocessed by the software supplied by the scanner manufacturers.

**Figure 1:**
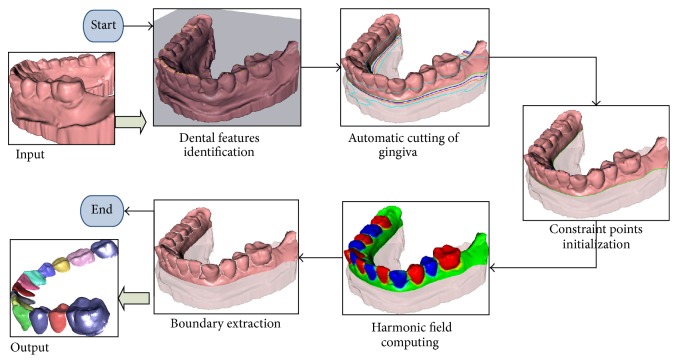
Block diagram of our proposed framework.

These dental mesh models are taken as input of the proposed framework as demonstrated in Figure 1, and the output results are segmented individual teeth.

As illustrated in the block diagram, teeth anatomical feature points and the occlusal plane, employed as the prior knowledge of following computation, are firstly identified (Section 3.1). Secondly, a rough locating procedure for teeth parts is performed and a cut plane is found to automatically remove the gingival region of the dental model (Section 3.2). Then, dental-targeted constraint points can be initialized to prepare for the harmonic field calculation. Once a harmonic field is generated, an optimal isoloop for each tooth can be extracted as a tooth boundary (Section 3.3).

### 3.1. Dental Features Identification

**Figure 2:**
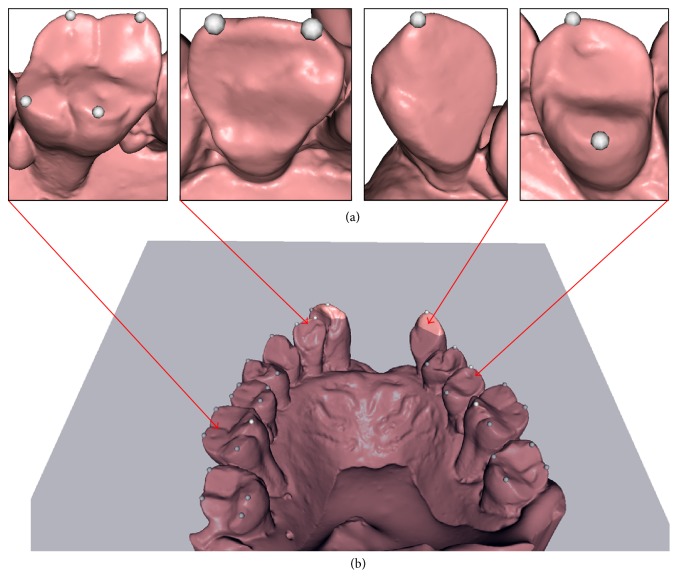
Automatically identified features of a dental model. Top row from left to right indicates anatomical feature points on molar, incisor, canine, and premolar; bottom row illustrates the occlusal plane.

We use a priori knowledge of feature points on human teeth and occlusal plane in our method. The feature points consist of cusps on the canines, premolars, and molars and point to the end of the incisal edge on incisors. Identification of these points and the occlusal plane is a fundamental dental process. A robust, computer-aided, automatic identification method [27] is used in our framework to identify them, as demonstrated by white spheres and a blue plane in Figure 2.

The inspection spoke-based strategy [20] is then employed to automatically separate these teeth feature points into different groups. This approach first fits a curve of the tooth-based dental arch and then detects inspection spokes along the dental arch. Instead of using the inspection spokes to do tooth segmentation, which may lead to unsatisfactory cut results, we utilize them to separate those feature points into different groups (each group corresponding to one tooth).

In addition, these feature point groups are arranged in order along the direction of the dental arch. And we further classify them into two bigger point sets, that is, one feature points set, *Ω*_1_, consisting of groups with odd indexes of 1,3, 5,… and the other set, *Ω*_2_, consisting of alternating groups with even indexes of 2,4, 6,…. These two feature point sets will be employed as part of constraints in the computation of the dental-targeted harmonic field.

### 3.2. Automatic Cutting of Gingiva

We seek a cutting plane to roughly separate the teeth parts from the gingiva region to accelerate following harmonic field computation. The basic idea is similar to earlier methods [1], but we have made it more efficient. Initially, the plane is located at the position of the occlusal plane and then moved iteratively towards the bottom of the dental model along the normal direction. The distance moved in each step is a small constant value, *d* (we noticed that *d* = 1 mm meets the requirement of almost all dental models in our experiments). During the movement, each plane *P*_*i*_ (1 ≤ *i* ≤ *N*, *N* is the count of move steps) will intersect with dental mesh and generate one or more intersecting loops.

**Figure 3:**
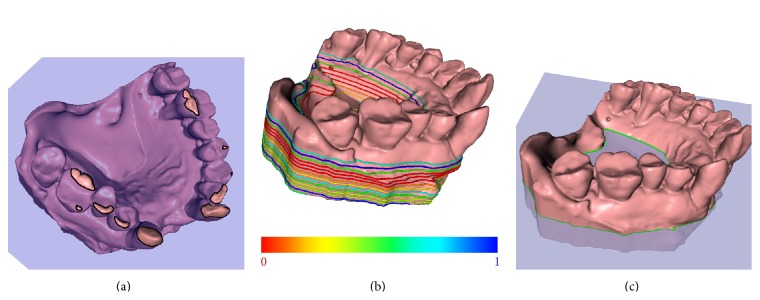
Steps of gingiva cutting. Images from left to right illustrate (a) the inappropriate cutting when multiple intersection loops are acquired; (b) the acquisition of only one intersection loop; (c) the desirable cutting attained when the variance energy stops decreasing.

At the beginning, a multiple-loop intersection will be detected as shown in Figure 3(a), which implies that the plane is intersecting with the teeth parts or outliers. The iteration is continued until only one-loop intersection is acquired, as the first meaningful loop shown in Figure 3(b). Afterwards, for each *P*_*i*_, the variance energy of minimum principal curvature, *ϕ*_*i*_, is used to evaluate the intersection:

(5) $$\begin{array}{c} \phi_{i}=\frac{1}{n-1}\overset{n}{\underset{k=1}{\sum }}{\left(c_{\min,k}-c_{\min,\text{avg}}\right)}^{2}, \end{array}$$

where *n* is the number of mesh points on the intersection loop, *c*_min⁡⁡,*k*_ denotes the minimum principal curvature of point *k*, and *c*_min⁡⁡,avg_ indicates the average minimum principal curvature of all points.

When all one-loop intersections are found, their corresponding variance energies are linearly mapped to axes 0, 1. The results are shown as colors ranging from red to blue in Figure 3(b). The normalized variance energy of each, *P*_*i*_, is utilized to select the best cutting plane because we notice that the energy is high at the teeth parts (larger than 0.5 generally) and decreases towards the gingiva region. Based on this observation, we select the cutting plane by checking the normalized variance, which is less than 0.5 in each step, until it stops decreasing. In the extreme situation, the movement stops when there is no intersection of the plane with the dental model; in this case, we use the last one-loop intersection plane as the cutting plane.

Finally, useless dental parts (e.g., the transparent gingiva region in Figure 3(c)) are clipped out using the cutting plane. In addition, the mesh points on the loop are recorded as part of constraints in the following harmonic field computation (see Section 3.3).

Procedures described in this section are valuable for high-precision dental meshes because large percentages of useless mesh can be removed to relieve the burden of harmonic field computation. Although general mesh decimation processes can be also used to reduce complexity of mesh, along with the global decimation quality of teeth surfaces will receive damage as well.

### 3.3. Harmonic Field Calculation

We adopted the basic idea of utilizing harmonic field for tooth boundary identification [24], which makes use of a special weighting scheme, given by

(6) $$\begin{array}{c} w_{ij}^{*}=\frac{\gamma_{ij}\left(\text{cot}\alpha_{ij}+\text{cot}\beta_{ij}\right)}{2}. \end{array}$$

The properties of this kind of harmonic field can be summarized as follows, which is the reason we chose it for the tooth partition.

*(i) Smoothness*. As a method derived from classic cotangent-weighting scheme, the harmonic field successfully inherits the smooth transition property. That is, the transitions of vertex scalars from minimum to maximum Dirichlet boundary values are stable and smooth in both concave and nonconcave regions. 

*(ii) Shape-Awareness*. The harmonic field has strong awareness of concave creases and seams. Therefore, the uniformly sampled isolines, which are dense at concave regions, can naturally form the candidates of partition boundaries.

**Figure 4:**
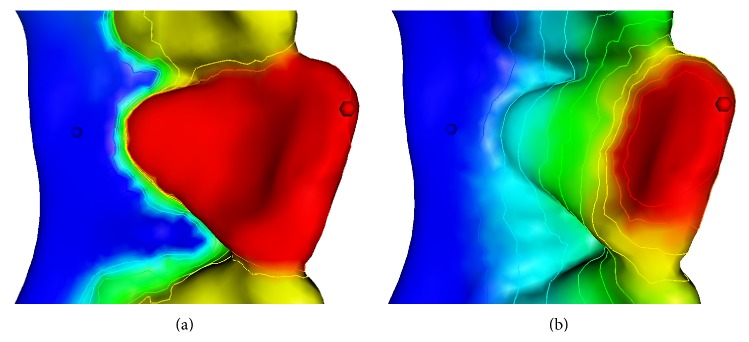
Harmonic fields under different weighting schemes. The images demonstrate harmonic field under (a) the special weighting scheme and (b) cotangent-weighting scheme, respectively.

Figure 4 demonstrates differences between two harmonic fields by mapping field scalars, whose values range from 0 to 1 and the colors range from red to blue.  Figure 4(a) shows the harmonic field using the special weighting scheme, while Figure 4(b) shows the harmonic field with the same constraints but uses the standard cotangent-weighting scheme. Uniformly sampled isolines on mesh are also extracted and colored to indicate field variance.

Our new dental-targeted segmentation framework is different from previous tooth-targeted study [24] in two major aspects. The first one is that the constraints of dental harmonic field are automatically identified as proposed above. And the second one is our special designed assignment of constraints for dental teeth segmentation, as introduced in the following, which is the key to segment all teeth only once under a uniform harmonic field computation automatically.

For Dirichlet constraints of the dental-targeted harmonic field, there are two major considerations: (1) the method for choosing constraint points on dental meshes and (2) the value of constraint points assigned in harmonic field computation. For the first issue, we choose constraint points by taking a priori knowledge of human teeth into consideration. In other words, the teeth feature points sets, *Ω*_1_ and *Ω*_2_, and the mesh points set, *Ω*_3_, on the gingiva cutting plane are used as constraints.

**Figure 5:**
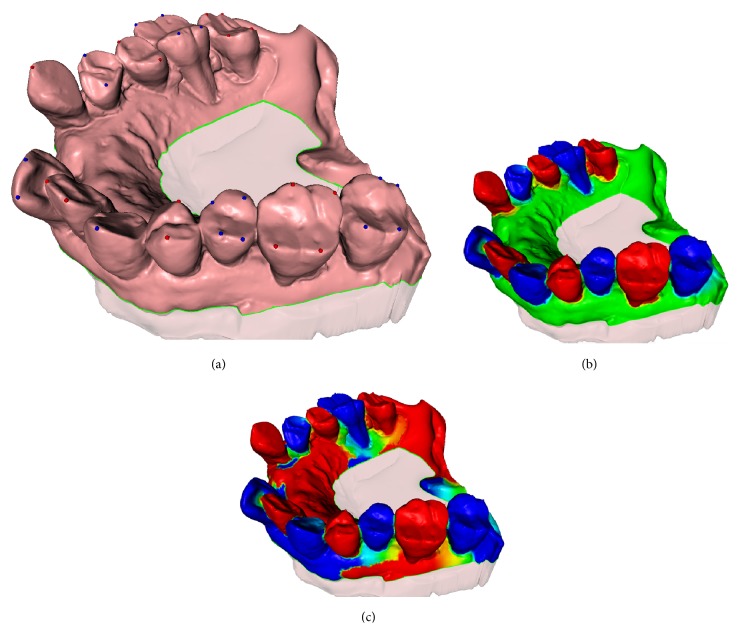
Assignment of constraint points on dental mesh for harmonic field computation. (a) Constraints assigned in our proposed method. (b) Resulting harmonic field with intersecting contour mesh points as constraints. (c) Resulting field without intersecting contour mesh points as constraints.

For the second issue, the maximum and minimum constraint values 1 and 0 are assigned to the teeth feature points sets, *Ω*_1_ and *Ω*_2_, separately. That is, feature points on the same tooth will have the same constraint value, either 0 or 1, but feature points on the neighbor teeth will have different constraint value, either 1 or 0, as demonstrated by red and blue spheres in Figure 5(a), respectively.

In addition, a middle constraint value of 0.5 is assigned to the mesh points set, *Ω*_3_, on the gingiva cutting plane (as green contour and points depicted in Figure 5(a)).

As demonstrated by Figures 5(b) and 5(c), the resulting harmonic field, using our assignment strategy of a priori knowledge guided constraints, has much better distinguishing tooth partition patterns for all teeth, compared to one without such middle constraint value, which is a conventional case in many other harmonic field-based methods.

According to the constraints assignment, entities of matrix *C* and vector *b*′ introduced above can be set solving 2. Specifically, if we denote points assigned with constraint values 0, 0.5, and 1 by *p*_min⁡_, *p*_mid_, and *p*_max⁡_ on mesh *M*, respectively, and put them into a list *S*, then *c*_*ij*_ ∈ *C* (1 ≤ *i* ≤ *n*, 1 ≤ *j* ≤ *m*) and *b*_*i*_ ∈ *b*′ (1 ≤ *i* ≤ *n*) can be given by the following equations, respectively:

(7) $$\begin{array}{c} c_{ij}=\left\{\begin{array}{cc} w, & \text{for}\;\;\left\{\left(i,j\right)\;|\;M\left(p_{i}\right)=j\right\},\\ 0, & \text{otherwise}, \end{array}\right. \end{array}$$

(8) $$\begin{array}{c} b_{i}=\left\{\begin{array}{cc} w, & \text{for}\;\;\left\{i\;|\;\text{Type}\left(p_{i}\right)=p_{\max}\right\},\\ 0.5w, & \text{for}\;\;\left\{i\;|\;\text{Type}\left(p_{i}\right)=p_{\text{mid}}\right\},\\ 0, & \text{for}\;\;\left\{i\;|\;\text{Type}\left(p_{i}\right)=p_{\min}\right\}, \end{array}\right. \end{array}$$

where *w* is a large constant value (1000 in our experiments), *n* and *m* denote number of vertexes in *S* and *M*, respectively, and *p*_*i*_ (0 ≤ *i* ≤ *n* − 1) is an element of *S*, whose index is *i*. *M*(*p*_*i*_) denotes the index of *p*_*i*_ in *M*, Type(*p*_*i*_), and returns the type of *p*_*i*_.

Modern sparse Cholesky factorization and modification software package [28, 29] are used to solve the linear system (i.e., 2) efficiently, which results in the dental-targeted harmonic field showed in Figure 5(b).

**Figure 6:**
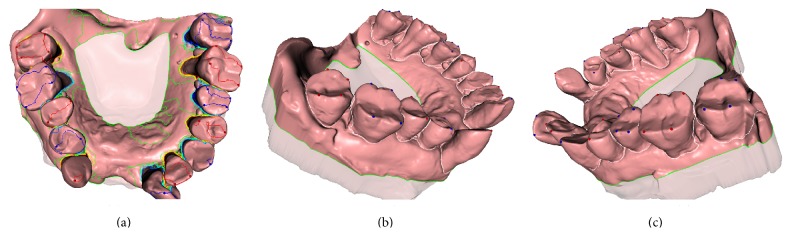
Tooth boundaries identification: (a) isoloops evenly extracted from generated harmonic field; (b and c) two perspectives of the extracted optimal isoloops as the tooth boundaries.

With our objective almost accomplished, all that is left to sample and uniformly extract is a number of isolines from the harmonic field, as shown by colored loops in Figure 6(a), and select optimal isoloops as the tooth boundaries (e.g., white loops in Figures 6(b) and 6(c)). And we use the voting strategy proposed in previous work [5] to automatically select the best isoloop.

## 4. Experiments and Results

**Figure 7:**
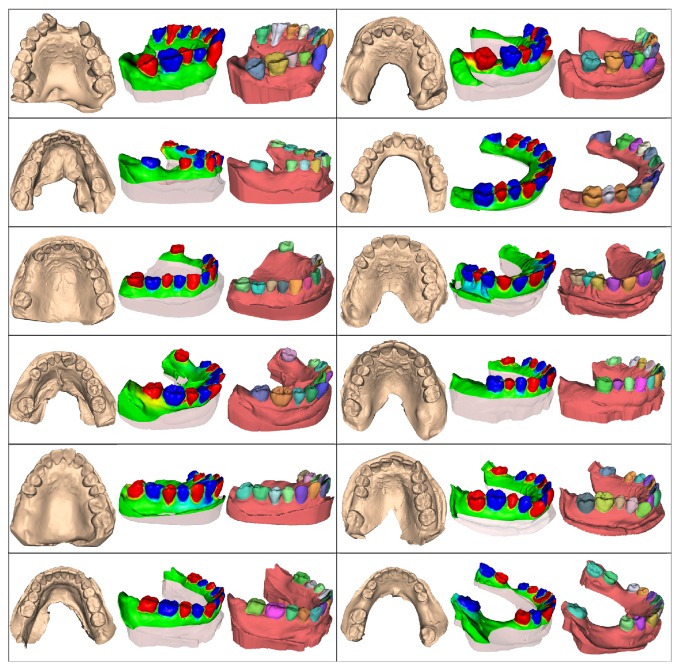
The segmentation results of our approach on various dental meshes with crowding problems.

We have tested our approach on 60 dental mesh models (low and up jaw) of varying complexity. The datasets included laser scans of plaster models obtained from different commercial scanners. Our approach was performed with reasonable accuracy on almost all of these models.  Figure 7 illustrates 12 of these dental models, some of which show teeth with severe crowding problems, while others may be absent from the jaw. For each of the 12 models in Figure 7, three images are used to illustrate states during the partition, namely, (1) the input original mesh, (2) the clipped dental mesh attached with dental-targeted harmonic field, and (3) the segmented nonteeth part (colored with red) and individual teeth (with other colors) as output.

**Figure 8:**
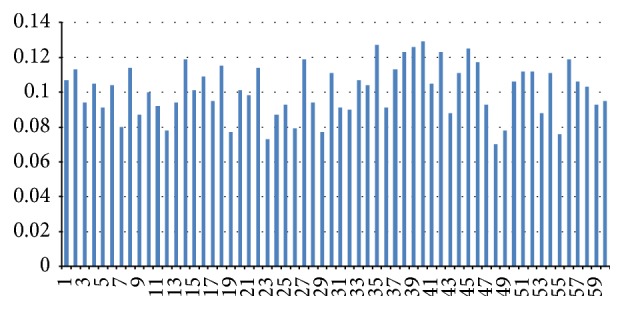
The mean errors of our tooth segmentation results compared to manually labeled ground truth of all 60 models. The horizontal axis denotes 60 cases in our experiments and the vertical axis denotes the mean errors, correspondingly.

To provide a “ground truth” dental segmentation benchmark for quantitative evaluation, we asked two dentists, each with the necessary training and sufficient practice time, to identify the boundary of each tooth manually on all experimental models. For each tooth, all marked contours from the two dentists were averaged to produce a ground truth. The boundaries of our segmented teeth were then compared with the ground truth results using mean errors, as shown in Figure 8. The average mean error of our approach within the 60 models is about 0.1 mm, which was approved by the dentists.

**Figure 9:**
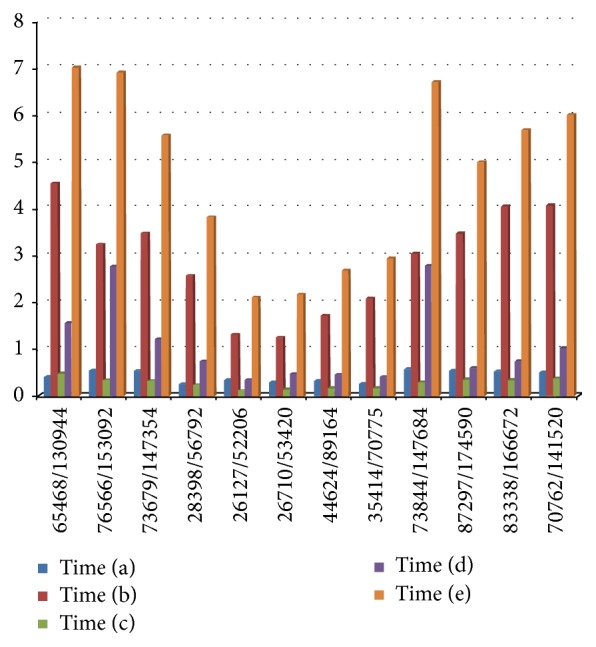
Time statistics of our dental mesh segmentation. The timing is recorded in seconds for (a) dental base cutting, (b) harmonic field precomputing, (c) harmonic field updating, (d) boundary extraction, and (e) total time of dental segmentation. The horizontal axis illustrates different mesh model scales by number of mesh points and faces. The vertical axis illustrates the time consumption for procedures in the segmentation framework.

We also recorded the time consumed by our approach on different scales of dental models (measured with a number of mesh points and faces) during experiments, as shown in Figure 9, including the time for (a) dental base cutting, (b) harmonic field precomputing, (c) harmonic field updating, (d) boundary extraction, and (e) total time for dental segmentation. All experiments were carried out on a common PC with Intel Core Quad-Core Processor 2.67 GHz with 4 GB memory. The total segmentation time of one dental model in our experiments is usually less than 7 seconds. By contrast, the popular commercial software, “3Shape,” often takes many minutes of interaction to segment one model; the accompanying method [1] often takes 1 or 2 minutes.

## 5. Conclusions

For this paper, we studied the fundamental problem of automatically segmenting teeth in dental mesh models into individual tooth objects. With a specially designed weighting scheme and a strategy of a priori knowledge to guide the assignment of constraints, we built a novel dental-targeted harmonic field, which is able to segment all teeth only once under a uniform harmonic field computation automatically. This harmonic field is robust to various tooth shapes, complex malocclusion, and crowding problems and can guarantee closed tooth boundary extraction from dental mesh, unlike curvature-based methods needing complex connectivity and morphologic operations, in most cases.

Extensive experiments and quantitative analysis demonstrated the effectiveness of the method in terms of accuracy, robustness, and efficiency. We plan to integrate this convenient, dental segmenting algorithm in a computer-aided, 3D-orthodontic system that is suitable for deployment in clinical settings.
